# Biocompatibility and Wound-Healing Performance of an Amberjack (*Seriola quinqueradiata*) Skin-Derived Acellular Dermal Matrix in a Porcine Full-Thickness Wound Model

**DOI:** 10.3390/md24100360

**Published:** 2026-10-17

**Authors:** Keumyeon Kim, Honggeun Shin, Dogeon Yoon, Seong Uk Jang, Seongyeon Jo, Yong Suk Cho, Hong Kee Kim

**Affiliations:** 1 Medical Device Division, SCL Science Inc., Seoul 07282, Republic of Korea; reguluskky@naver.com (K.K.); hgshin@sclscience.com (H.S.); swjang@sclscience.com (S.U.J.); josy@sclscience.com (S.J.); 2 Burn Institute, Hangang Sacred Heart Hospital, Seoul 07247, Republic of Korea; hyeonyoon@hallym.ac.kr; 3 Burn Institute, Pohang St. Mary’s Hospital, Pohang 37661, Republic of Korea

**Keywords:** amberjack (*Seriola quinqueradiata*), fish skin, acellular dermal matrix, fish skin-derived biomaterial, wound healing, decellularization, porcine full-thickness wound model, marine biomaterial

## Abstract

Although fish skin-derived acellular dermal matrices (ADMs) are emerging non-mammalian wound matrices, species beyond cod and tilapia remain underexplored. In this study, we prepared an ADM from Japanese amberjack (*Seriola quinqueradiata*) skin and evaluated its physicochemical properties, biocompatibility, and wound-healing performance in comparison with a commercial cod skin-derived matrix (Kerecis Omega3 Wound) and untreated wounds. The amberjack skin-derived ADM (AFS) was produced through decellularization, decolorization, and freeze-drying and was characterized by residual dsDNA content, extracellular matrix composition, microstructure, swelling behavior, tensile properties, cytocompatibility (ISO 10993-5), rat subcutaneous implantation (ISO 10993-6), and a 42-day porcine full-thickness wound model. AFS showed efficient decellularization, with residual dsDNA reduced to 29.2 ± 7.6 ng/mg, and retained a collagen-rich extracellular matrix with asymmetric porous microarchitecture, rapid swelling, a hydrated tensile strength of 2.4 ± 0.4 MPa, and acceptable cytocompatibility. In porcine wounds, AFS significantly enhanced early re-epithelialization compared with untreated wounds on Days 7 and 11 and reduced early expansion of the periwound tattooed area, while barrier-related parameters normalized over time. During remodeling, AFS-treated wounds showed greater total collagen and type I collagen deposition than untreated wounds, with no significant differences from the comparator. These findings support amberjack skin as a promising marine-derived source for resorbable wound-covering ADMs.

## 1. Introduction

Skin wounds caused by trauma, burns, surgical excision, diabetic foot ulcers (DFUs), pressure injuries, and venous leg ulcers (VLUs) continue to impose a substantial clinical and economic burden worldwide [1,2,3]. Global wound care expenditure reached USD 148.65 billion in 2022, and this burden is expected to escalate as populations age and the prevalence of diabetes and obesity continues to rise [1]. In the United States, Medicare expenditures for wound care are estimated to be between USD 28.1 billion and USD 96.8 billion annually [1,3]. Such wounds often require prolonged treatment and can lead to infection, fluid loss, pain, and functional impairment [2,4]. In wounds with substantial dermal loss, spontaneous healing frequently proceeds through contraction and fibrotic scar formation rather than the regeneration of normal skin architecture, resulting in compromised mechanical function and poor cosmetic outcomes [4,5,6].

Although autologous skin grafting remains the standard of care for deep and extensive wounds, its clinical use is limited by donor-site morbidity, additional surgical burden, pain, and restricted tissue availability [7,8]. Allogeneic grafts and other cellular or tissue-based products have been introduced to address some of these limitations, but concerns regarding immunologic responses, variable long-term performance, high cost, and incomplete restoration of native skin structure and function remain unresolved [8,9,10]. Acellular dermal matrices (ADMs), primarily derived from mammalian sources such as porcine, bovine, and human cadaveric tissues, have gained widespread adoption as alternatives that preserve native extracellular matrix (ECM) architecture while removing immunogenic cellular components [11,12]. However, the intensive decellularization and viral inactivation procedures required for mammalian-derived ADMs can alter ECM ultrastructure and remove biologically active components, including glycosaminoglycans, soluble collagens, and matrix-bound growth factors, thereby reducing the overall biological complexity of the resulting matrix [12,13,14]. Additionally, mammalian-derived materials require rigorous control of potential zoonotic pathogens, including porcine endogenous retroviruses and bovine spongiform encephalopathy-associated prions, while their porcine or bovine origin may also limit cultural or religious acceptability [15,16]. Therefore, there is a persistent need for off-the-shelf skin substitute materials that are biocompatible, bioactive, readily available, conformable to irregular wound beds, and capable of supporting durable dermal reconstruction rather than temporary wound coverage alone.

In this context, fish skin-derived ADMs have attracted increasing attention as non-mammalian skin substitutes [17]. Their collagen-rich dermal ECM and porous fibrillar architecture provide a favorable microenvironment for cell attachment and support angiogenesis, collagen deposition, and tissue remodeling [17,18,19]. Compared with mammalian-derived materials, fish skin-derived ADMs can be prepared using milder processing methods, thereby preserving native structural organization and molecular complexity to a greater extent [17,19]. Furthermore, because fish are phylogenetically distant from mammals and lack the highly immunogenic α-Gal epitope found in many non-primate mammalian tissues, these matrices present lower immunologic barriers in xenogeneic applications [17,20,21]. Additionally, fish skin contains omega-3 polyunsaturated fatty acids (PUFAs), which have been proposed to contribute to anti-inflammatory and pro-resolving responses during wound healing when retained in minimally processed fish skin grafts [22,23]. The clinical potential of fish skin-derived ADMs has been most extensively demonstrated for a commercial cod skin-derived ADM (Kerecis Omega3 Wound, Kerecis, Isafjordur, Iceland) [24,25,26], and tilapia skin-derived matrices have also shown favorable results in both preclinical and clinical wound repair [18,19,27]. However, current evidence remains concentrated on a small number of species, particularly cod and tilapia. Fish species differ in habitat temperature and skin structure, and such differences have been associated with variation in ECM composition, collagen thermal stability, and degradation behavior [18,28]. How these species-dependent characteristics translate into the performance of intact fish skin-derived ADMs, however, remains insufficiently characterized.

Japanese amberjack (*Seriola quinqueradiata*; hereafter, amberjack), an economically important marine fish widely distributed in Northeast Asia [29,30], represents a regionally relevant yet underexplored biomaterial source. According to the Food and Agriculture Organization of the United Nations (FAO), mariculture production of amberjack, predominantly in Japan and the Republic of Korea, has historically exceeded 150,000 tonnes annually, generating substantial quantities of collagen-rich skin as a processing byproduct [31,32]. Valorizing this underutilized biomass as a biomedical material could broaden the range of fish skin-based wound care products while contributing to a circular bioeconomy [15,33]. Recently, Bui et al. incorporated soluble fractions of decellularized amberjack skin into a hybrid bioink, demonstrating enhanced cell viability, fibroblast infiltration, and the formation of bilayered skin-like constructs in vitro [34]. However, that study was limited to solubilized components and did not evaluate the structural integrity or in vivo wound-healing potential of an intact, solid-state amberjack-derived ADM.

In the present study, we prepared an ADM from amberjack skin through a decellularization and decolorization process and characterized its residual dsDNA content, biochemical composition, microstructure, porosity, swelling behavior, and tensile properties. Cytocompatibility and local tissue response were evaluated in accordance with ISO 10993-5 [35] and ISO 10993-6 [36], the latter over an eight-week subcutaneous implantation period that also allowed assessment of in vivo degradation. The matrix was then applied to full-thickness excisional wounds in a porcine model and followed for 42 days alongside a commercially available cod skin-derived ADM (Kerecis Omega3 Wound) and untreated wounds, with re-epithelialization, wound contraction, non-invasive barrier function (transepidermal water loss and stratum corneum hydration), and histological collagen remodeling as endpoints. Rather than seeking to demonstrate superiority over existing products, our aim was to determine whether an amberjack skin-derived ADM meets established safety criteria and supports wound healing to a degree comparable with a clinically used fish skin ADM. To our knowledge, this is the first preclinical evaluation of an intact, solid-state amberjack skin-derived ADM, and the first head-to-head comparison of a newly developed fish skin ADM with an established commercial fish skin product in a porcine full-thickness wound model.

## 2. Results

### 2.1. Preparation and Characterization of the Amberjack Skin-Derived ADM

**Figure 1:**
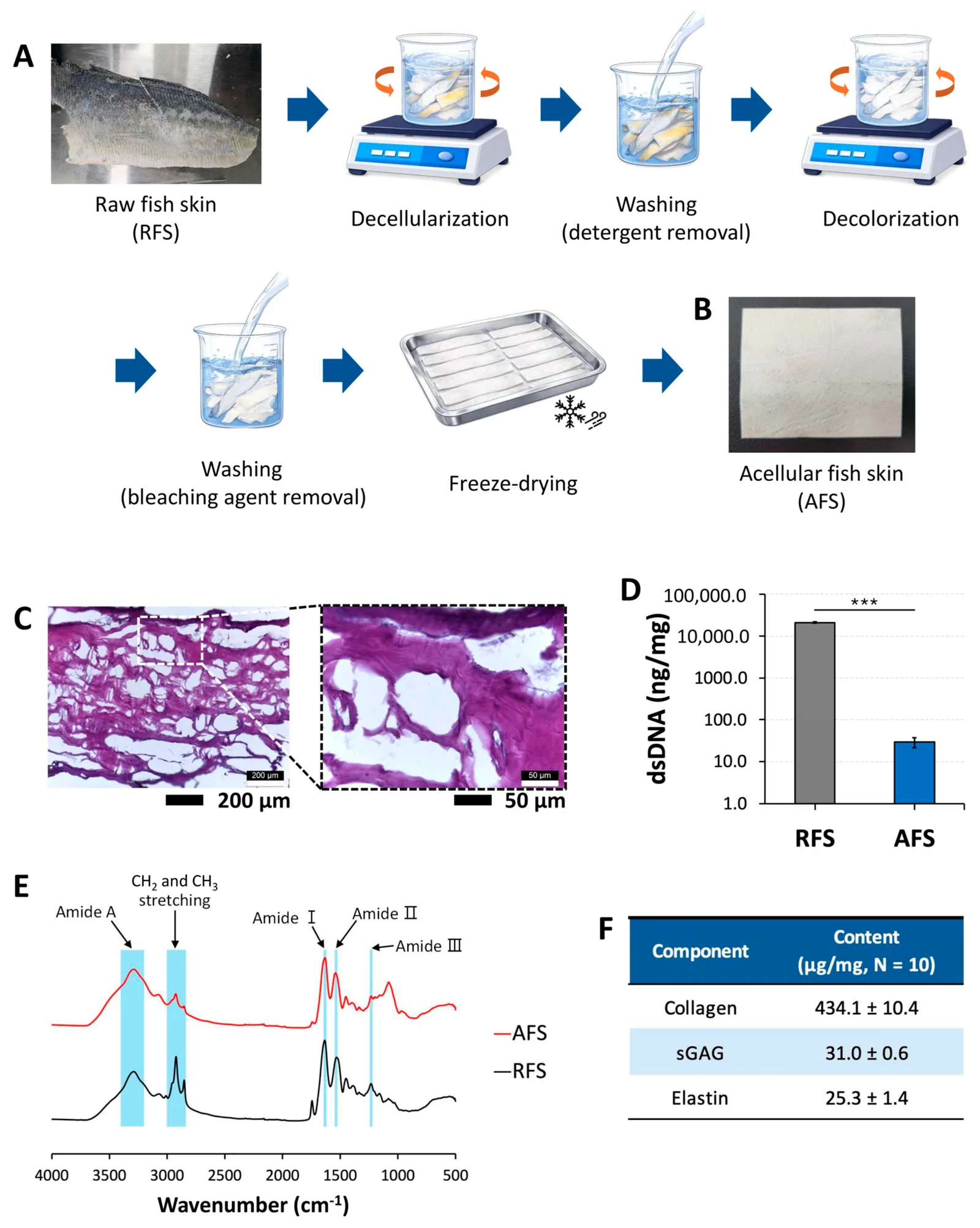
Preparation and physicochemical characterization of the amberjack skin-derived acellular dermal matrix (ADM). (**A**) Schematic of the overall preparation process, comprising decellularization (0.5% SDS, ≥12 h), washing for detergent removal, decolorization (ammonium persulfate/sodium metasilicate/H_2_O_2_, 1.5 h), washing for bleaching agent removal, and freeze-drying (≥48 h); detailed conditions are provided in Section 4.1. (**B**) Appearance of the final acellular fish skin (AFS) sheet. (**C**) Representative hematoxylin and eosin (H&E)-stained section of AFS showing a porous, interconnected collagenous network devoid of visible nuclei; the boxed region is shown at higher magnification on the right (scale bars: 200 µm, left; 50 µm, right). The comparison with RFS was performed quantitatively by dsDNA content (**D**). (**D**) Residual double-stranded DNA (dsDNA) content in raw fish skin (RFS) and AFS, expressed as ng/mg dry weight (mean ± SD, *n* = 3; note the logarithmic scale of the *y*-axis, *** *p* < 0.001). (**E**) Attenuated total reflectance–Fourier transform infrared (ATR-FTIR) spectra of AFS (red) and RFS (black), with the characteristic amide A, CH_2_ and CH_3_ stretching, and amide I, II, and III bands highlighted. (**F**) Biochemical composition of AFS, showing collagen, sulfated glycosaminoglycan (sGAG), and elastin contents expressed as µg/mg dry weight (mean ± SD, *n* = 10).

An amberjack skin-derived ADM was prepared through a sequential process comprising decellularization, detergent removal, decolorization, bleaching agent removal, and freeze-drying (Figure 1A). Raw fish skin (RFS), which initially exhibited a dark and pigmented appearance, was transformed after processing into a soft, white, and pliable acellular fish skin (AFS) sheet while retaining an intact sheet-like form (Figure 1B).

Decellularization was assessed histologically in the final AFS and quantitatively by comparison with RFS. H&E staining of the final AFS showed a porous, interconnected fibrous ECM network with no visible nuclei or intact cellular structures (Figure 1C). Consistent with this observation, residual dsDNA content decreased from 21,286.0 ± 1003.0 ng/mg in RFS to 29.2 ± 7.6 ng/mg in AFS, corresponding to an approximately 99.9% reduction (*p* < 0.001; Figure 1D). The residual dsDNA content of the AFS was below the commonly applied decellularization criterion of 50 ng/mg dry weight [12].

The preservation of the collagen-associated chemical structure after decellularization was evaluated by ATR-FTIR spectroscopy (Figure 1E). The characteristic collagen-associated amide bands, including amide A (3290 cm^−1^), amide I (1640 cm^−1^), amide II (1540 cm^−1^), and amide III (1240 cm^−1^), were observed in both RFS and AFS, with comparable peak positions and band shapes [18,37,38]. A relative difference between the two spectra was observed in the C–H stretching region from 2800 to 3000 cm^−1^, which is primarily associated with symmetric and asymmetric CH_2_ and CH_3_ stretching vibrations.

The biochemical composition of the final AFS was quantified in terms of its principal ECM components (Figure 1F). Collagen was the predominant measured constituent, with a content of 434.1 ± 10.4 µg/mg dry weight. The AFS also contained 31.0 ± 0.6 µg/mg of sulfated glycosaminoglycans (sGAGs) and 25.3 ± 1.4 µg/mg of elastin. Collectively, these results demonstrated effective removal of cellular material and the production of an intact, collagen-rich amberjack skin-derived matrix containing measurable amounts of non-collagenous ECM components.

### 2.2. Microstructural, Mechanical, and Biocompatibility Characteristics of the Amberjack Skin-Derived ADM

**Figure 2:**
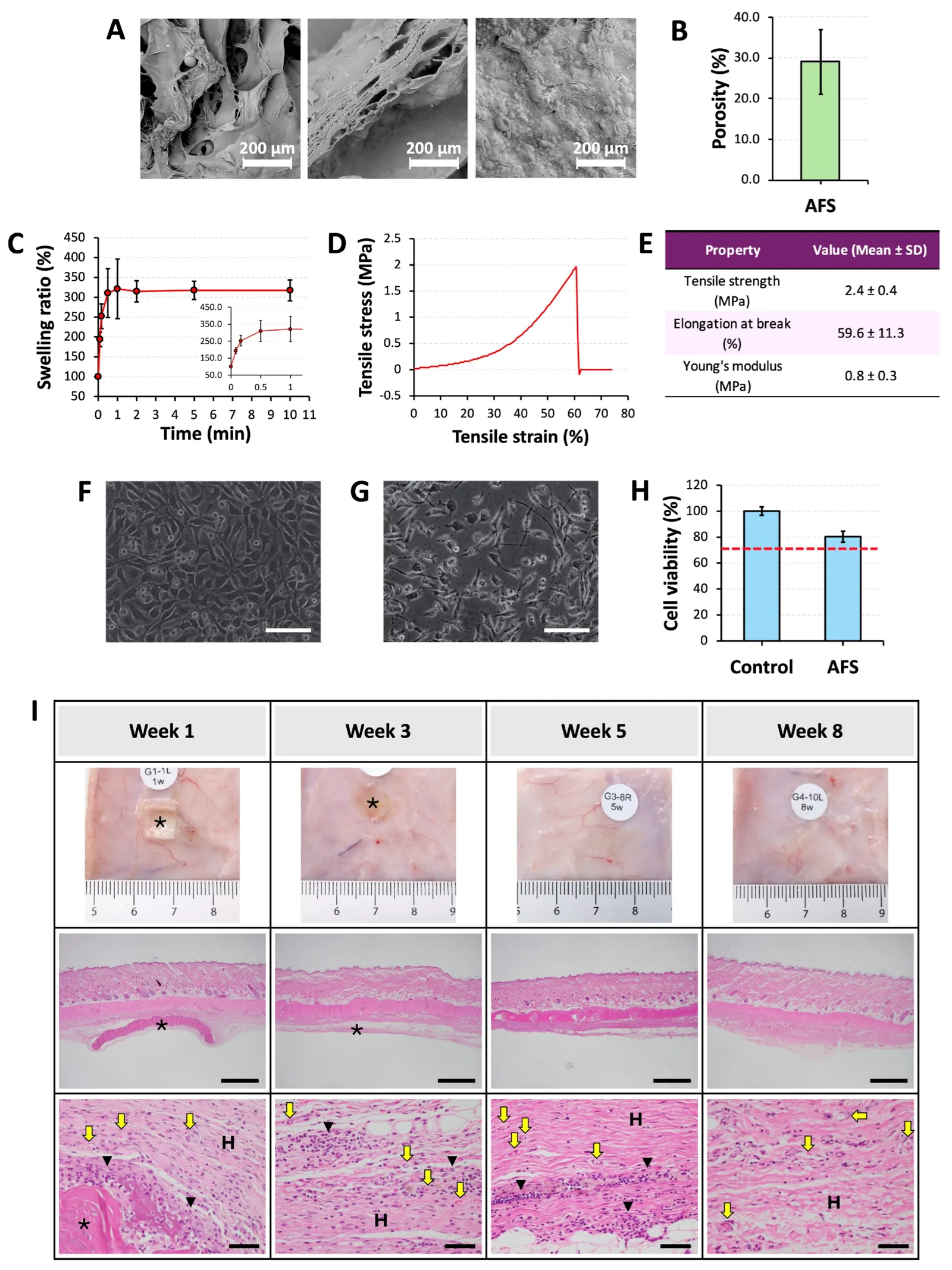
Microstructural, mechanical, and biocompatibility characteristics of the amberjack skin-derived ADM (AFS). (**A**) Representative scanning electron microscopy (SEM) images of the AFS showing, from left to right, the inner (dermal side) surface, the cross-section, and the outer (scale side) surface. Scale bars: 200 µm. (**B**) Bulk porosity of the AFS determined through the ethanol displacement method. (**C**) Swelling behavior of the AFS in normal saline over 10 min; the inset shows the first 60 s on an expanded time scale. (**D**) Representative tensile stress–strain curve of the hydrated AFS. (**E**) Tensile strength, elongation at break, and initial Young’s modulus (0–10% strain region) of the hydrated AFS determined by uniaxial tensile testing. (**F**,**G**) Representative microscopic images of L929 fibroblasts exposed to control medium (**F**) or AFS extract (**G**), in accordance with ISO 10993-5. Scale bars = 100 µm. (**H**) Cell viability of L929 fibroblasts exposed to the AFS extract relative to the control. The red dashed line indicates the 70% cell viability threshold in ISO 10993-5 (**I**) Representative gross and histological images of the subcutaneous implantation sites at 1, 3, 5, and 8 weeks after AFS implantation in SD rats. Top row: gross appearance of the implantation sites. Asterisks (*) indicate the implanted AFS. Middle row: low-magnification images of H&E-stained sections (scale bar = 2 mm). Bottom row: high-magnification images of the implant–host interface (scale bar = 500 μm). Asterisks (*), residual AFS; black arrowheads, inflammatory cell infiltrates; yellow arrows, blood vessels; H, host-derived fibrous connective tissue. Data in (**B**,**C**,**E**), and (**H**) are presented as the mean ± standard deviation (SD) (*n* = 3). ADM, acellular dermal matrix; AFS, acellular fish skin.

The surface and cross-sectional microstructure of the AFS was examined by scanning electron microscopy (SEM). The AFS displayed marked structural asymmetry, with distinct morphological features between the two surfaces and the cross-section (Figure 2A). From left to right, the inner (dermal side) surface exhibited a highly porous, open network of loosely arranged collagen fibers with large interconnected pores. The cross-section revealed a stratified collagenous architecture in which layered fiber bundles were separated by void spaces distributed across the matrix thickness. In contrast, the outer (scale side) surface presented a comparatively dense and compact fibrillar organization with a rougher texture. The bulk porosity of the AFS, measured using the ethanol displacement method, was 29.1 ± 8.0% (Figure 2B).

The fluid-handling capacity of the AFS was assessed by measuring its swelling behavior in normal saline. The AFS absorbed fluid rapidly, reaching swelling ratios of 194.0 ± 18.2%, 252.3 ± 30.9%, and 310.3 ± 61.5% at 5, 10, and 30 s, respectively, and 321.3 ± 75.2% within 1 min, after which the ratio remained relatively stable for up to 10 min (Figure 2C). The matrix absorbed an amount of saline equivalent to approximately 3.2 times its dry weight and reached an apparent swelling equilibrium within 1 min of immersion.

The mechanical properties of the hydrated AFS were evaluated by uniaxial tensile testing. The representative stress–strain curve exhibited a characteristic J-shaped profile typical of collagen-rich soft tissues [39], with an initial low-stiffness toe region followed by a progressive increase in stress until failure (Figure 2D). The hydrated AFS showed a tensile strength of 2.4 ± 0.4 MPa, an elongation at break of 59.6 ± 11.3%, and an initial Young’s modulus of 0.8 ± 0.3 MPa determined from the 0–10% strain region (Figure 2E).

The in vitro cytocompatibility of the AFS was evaluated using an extract-based assay with L929 fibroblasts in accordance with ISO 10993-5. Cells exposed to the AFS extract retained a normal, well-spread fibroblastic morphology comparable to that of the control cells, with no apparent cytotoxic changes, such as cell rounding, detachment, or lysis (Figure 2F,G). Quantitatively, cells exposed to the AFS extract showed a viability of 80.3 ± 4.3% relative to the control group (Figure 2H), well above the 70% cytotoxicity threshold defined by ISO 10993-5 [35]. These results indicated that the AFS extract did not exhibit cytotoxic potential.

The in vivo biocompatibility and degradation behavior of the AFS were evaluated by subcutaneous implantation in Sprague Dawley (SD) rats for up to 8 weeks. No gross evidence of severe local adverse reactions, including abscess formation, necrosis, or extrusion, was observed at any of the examined time points. Macroscopically, the implanted AFS (asterisks) became progressively less distinct and increasingly integrated with the surrounding tissue over time (Figure 2I, top row). Histologically, the residual AFS (asterisks) was identified in the subcutaneous layer as a dense, eosinophilic layer at week 1 and remained discernible, albeit progressively less distinct, at weeks 3 and 5. By week 8, it could no longer be clearly distinguished from the adjacent host tissue (Figure 2I, middle row). At week 1, the AFS elicited a mild host response, with inflammatory cells (arrowheads) infiltrating the interface between the residual AFS and the surrounding host tissue. At weeks 3 and 5, the inflammatory infiltrates were reduced and confined to focal aggregates, and small blood vessels (yellow arrows) were observed within the host-derived fibrous connective tissue (H) surrounding the implantation site. By week 8, inflammatory cells were sparse, and the implantation site was occupied predominantly by fibrous connective tissue containing blood vessels, indicating progressive integration of the AFS with the host tissue (Figure 2I, bottom row).

### 2.3. Macroscopic Wound Healing and Skin Barrier Function Recovery in the Porcine Full-Thickness Wound Model

**Figure 3:**
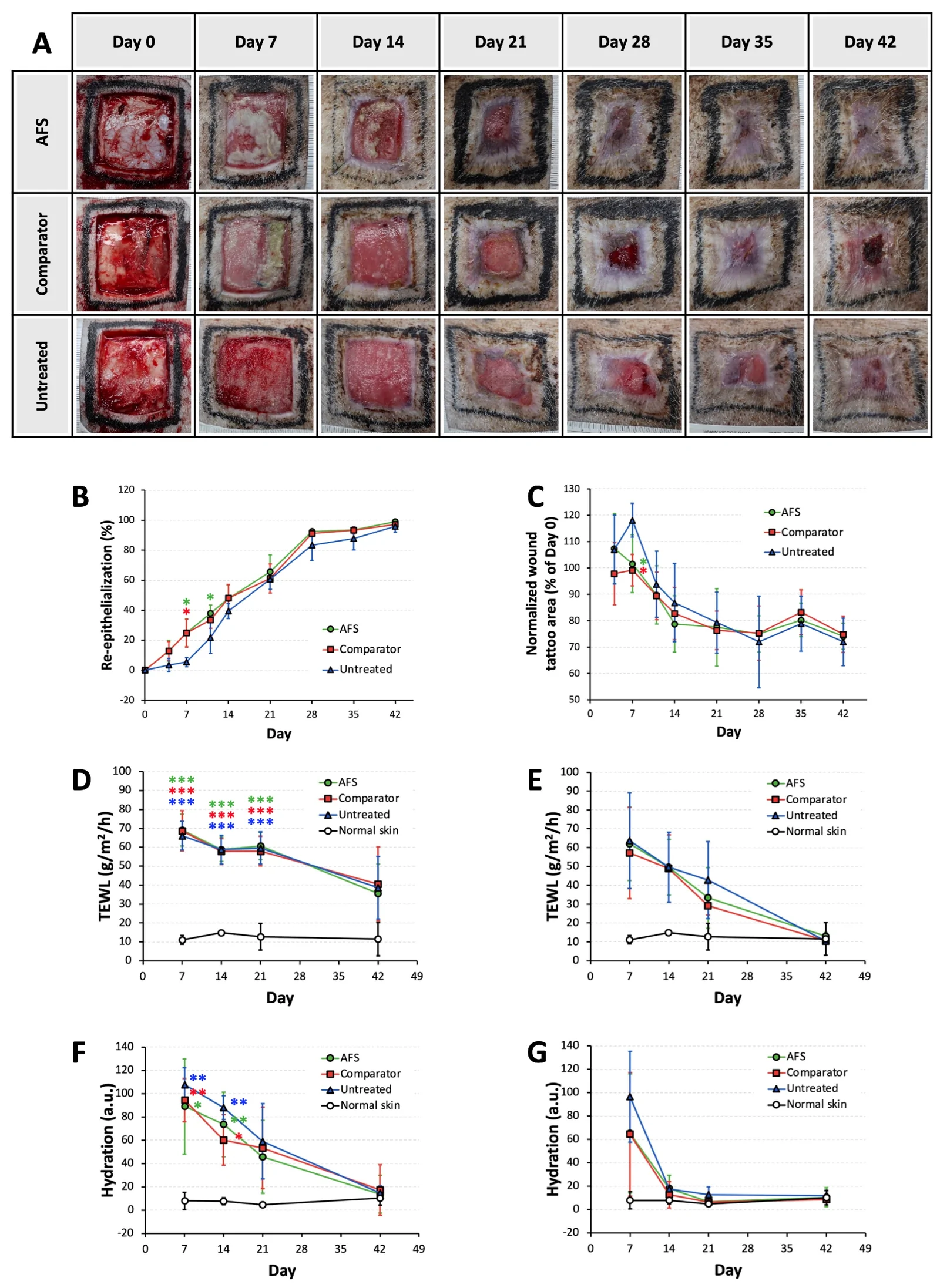
Macroscopic wound healing and skin barrier function recovery following application of the amberjack skin-derived ADM in a porcine full-thickness wound model. (**A**) Representative serial gross images of full-thickness excisional wounds treated with AFS, comparator (Kerecis Omega3 Wound), or left untreated, taken on Days 0, 7, 14, 21, 28, 35, and 42 after wounding. Each wound is surrounded by a tattooed reference square. Images in each row were obtained from the same wound in the same animal at all time points. The representative wound for each group was selected based on a qualitative review of overall wound-healing patterns. (**B**) Re-epithelialization (%), calculated as the reduction in open wound area relative to the initial wound area. (**C**) Normalized wound tattoo area, expressed as a percentage of the Day 0 value after normalization to the non-wounded control tattoo to correct for growth-related changes in skin surface area. (**D**,**E**) Transepidermal water loss (TEWL) measured at (**D**) the wound center and (**E**) the wound edge on Days 7, 14, 21, and 42. (**F**,**G**) Stratum corneum hydration measured at (**F**) the wound center and (**G**) the wound edge on Days 7, 14, 21, and 42. In (**D**–**G**), values from non-wounded normal skin are shown as a reference baseline. Data are presented as the mean ± SD (*n* = 4 wounds per group; normal skin, *n* = 2). For wound-edge hydration on Day 14, values from all six wounds of one animal (pig #3) were unavailable because of a recording error and were treated as missing data (*n* = 2 wounds per group on that day). In (**B**,**C**), colored asterisks indicate significant differences in the correspondingly colored group versus the untreated group (green, AFS; red, comparator; * *p* < 0.05). In (**D**,**F**), asterisks denote significant differences in the wounded groups (green, AFS; red, comparator; blue, untreated) versus normal skin (* *p* < 0.05, ** *p* < 0.01, *** *p* < 0.001). AFS, acellular fish skin; ADM, acellular dermal matrix; a.u., arbitrary units.

Six full-thickness excisional wounds were created on the dorsum of each pig and randomly assigned to the AFS, comparator (Kerecis Omega3 Wound), or untreated control, with two wounds allocated to each group per animal (*n* = 4 wounds per group). Serial gross images showed progressive wound closure in all groups over the 42-day observation period (Figure 3A). Both the AFS and the comparator remained adherent to the wound bed during the first week and were no longer macroscopically distinguishable thereafter.

Re-epithelialization was quantified from serial wound images based on the reduction in macroscopically open wound area relative to the initial wound area (Figure 3B). All three groups showed a progressive increase in re-epithelialization over time. During the early healing phase, both material-treated groups showed significantly greater re-epithelialization than the untreated group. On Day 7, re-epithelialization reached 25.0 ± 9.2% in the AFS group and 24.8 ± 9.2% in the comparator group, compared with 5.5 ± 3.1% in the untreated group (*p* < 0.05 for both comparisons versus untreated). On Day 11, re-epithelialization in the AFS group remained significantly higher than that in the untreated group (37.9 ± 5.5% vs. 21.7 ± 10.4%; *p* < 0.05), whereas the comparator group showed 33.4 ± 5.3% and was not significantly different from the untreated group. From Day 14 onward, re-epithelialization values converged among the three groups. By Day 42, the values reached 99.1 ± 0.3%, 97.2 ± 2.9%, and 95.8 ± 3.7% in the AFS, comparator, and untreated groups, respectively, with no significant differences among the groups. Complete macroscopic wound closure was first observed on Day 28 in one of four AFS-treated wounds, whereas the earliest complete closure in the comparator and untreated groups was observed on Day 35. No significant differences in re-epithelialization were observed between the AFS and comparator groups at any time point.

Periwound skin contraction was assessed by measuring the tattooed area surrounding each wound after normalization to a non-wounded control tattoo to account for growth-related changes in skin surface area (Figure 3C). On Day 7, the normalized wound tattoo area increased to 118.1 ± 11.0% of its Day 0 value in the untreated group. In contrast, the corresponding values remained close to baseline in the AFS and comparator groups (101.6 ± 6.5% and 99.2 ± 5.9%, respectively) and were significantly lower than those in the untreated group (*p* < 0.05 for both comparisons). From Day 11 onward, the normalized wound tattoo area progressively decreased in all groups. By Day 42, it had decreased to 74.1 ± 9.0%, 74.8 ± 6.9%, and 71.9 ± 4.9% in the AFS, comparator, and untreated groups, respectively, corresponding to an approximately 25–28% reduction from Day 0. No significant differences among the groups were observed at this time point.

Recovery of skin barrier-related physiological properties was evaluated non-invasively by measuring transepidermal water loss (TEWL) at the wound center and wound edge (Figure 3D,E). At the wound center, TEWL was significantly higher in all wounded groups than in non-wounded normal skin from Day 7 to Day 21 (*p* < 0.001) and decreased thereafter. By Day 42, TEWL values were 35.8 ± 15.2, 40.6 ± 19.6, and 38.6 ± 16.5 g/m^2^/h in the AFS, comparator, and untreated groups, respectively, and no significant differences from normal skin were detected (Figure 3D). At the wound edge, TEWL decreased earlier than at the wound center. On Day 21, TEWL values were 33.3 ± 16.0, 29.1 ± 4.9, and 42.8 ± 20.4 g/m^2^/h in the AFS, comparator, and untreated groups, respectively, without significant differences among the wounded groups. By Day 42, no significant differences between the wound edge and normal skin were detected in any group (Figure 3E). No significant differences in TEWL were detected among the AFS, comparator, and untreated groups at any time point.

Stratum corneum hydration showed a similar trend toward normalization over time (Figure 3F,G). At the wound center, hydration values were significantly higher than those of normal skin on Days 7 and 14 in all wounded groups (*p* < 0.05 or *p* < 0.01). From Day 21 onward, no significant differences from normal skin were detected (Figure 3F). At the wound center, hydration values in the AFS group were 89.0 ± 41.0, 73.6 ± 27.7, and 45.8 ± 31.3 a.u. on Days 7, 14, and 21, respectively, compared with 107.8 ± 14.7, 88.0 ± 10.3, and 59.0 ± 32.3 a.u. in the untreated group. These differences were not statistically significant. At the wound edge, hydration values decreased earlier, and no significant differences from normal skin were detected from Day 14 onward in any group (Figure 3G). No significant differences in hydration were observed among the AFS, comparator, and untreated groups at any time point.

### 2.4. Histological Evaluation of Epidermal Regeneration and Wound Remodeling

**Figure 4:**
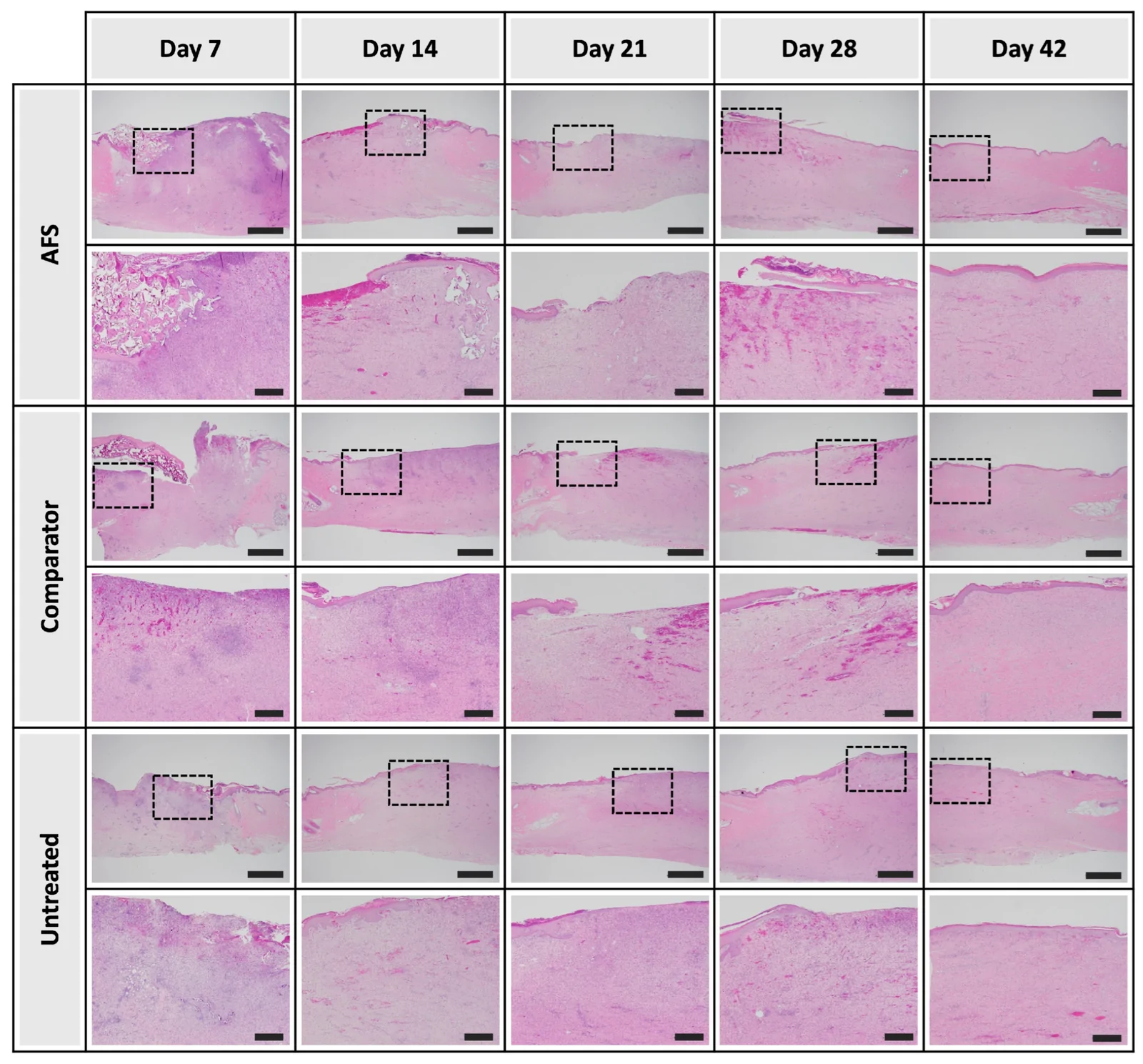
Histological evaluation of wound healing by H&E staining in the porcine full-thickness wound model. Representative H&E-stained sections of wounds treated with AFS, comparator (Kerecis Omega3 Wound), or left untreated, on Days 7, 14, 21, 28, and 42 after wounding. For each group and time point, the upper panel shows a low-magnification overview of the wound (original magnification 12.5×; scale bar, 2 mm), and the lower panel shows a higher-magnification view of the boxed region (dashed outline) at the wound surface (original magnification 40×; scale bar, 500 µm). AFS, acellular fish skin.

Histological evaluation was performed using H&E staining to assess epithelial regeneration, granulation tissue formation, vascularization, inflammatory cell infiltration, and wound-bed remodeling (Figure 4 and Supplementary Figure S1). Histological samples collected on Days 7 to 28 were obtained from a single animal designated for histopathological analysis and were therefore evaluated descriptively, whereas Day 42 samples comprised four wounds per group.

On Day 7, all groups showed granulation tissue formation, hemorrhagic changes, prominent vascular structures, and dense inflammatory cell infiltration. Partial epithelial coverage was observed in the AFS and comparator groups, whereas epithelial coverage appeared less advanced in the untreated group. Small amounts of residual foreign material accompanied by localized hemorrhagic changes were observed in some material-treated wounds. No foreign-body granulomatous reaction was identified.

From Day 14 to Day 28, progressive epithelial extension and wound-bed remodeling were observed in all groups. Angiogenesis remained prominent during this period, and granulation tissue formation and fibroplasia were consistently observed across the AFS, comparator, and untreated groups. Inflammatory cell infiltration gradually decreased over time in the AFS-treated wounds, with scores decreasing from severe infiltration on Day 7 to mild levels by Day 42. A similar temporal decrease in inflammatory response was observed in the comparator and untreated groups.

By Day 42, all groups showed extensive epithelial regeneration and remodeling of the wound bed. The epithelial status scores were 3.0 ± 0.0, 2.8 ± 0.5, and 2.5 ± 0.6 in the AFS, comparator, and untreated groups, respectively. Angiogenesis scores were 2.8 ± 0.5, 2.8 ± 0.5, and 3.0 ± 0.0 in the AFS, comparator, and untreated groups, respectively. The corresponding scores for granulation tissue formation and fibroplasia were 3.5 ± 1.3, 3.3 ± 1.3, and 4.3 ± 1.0. Inflammatory cell infiltration scores were 1.5 ± 0.6 in all three groups. No statistically significant differences among the groups were detected for any of the scored parameters at Day 42.

### 2.5. Collagen Deposition and Dermal Matrix Remodeling

**Figure 5:**
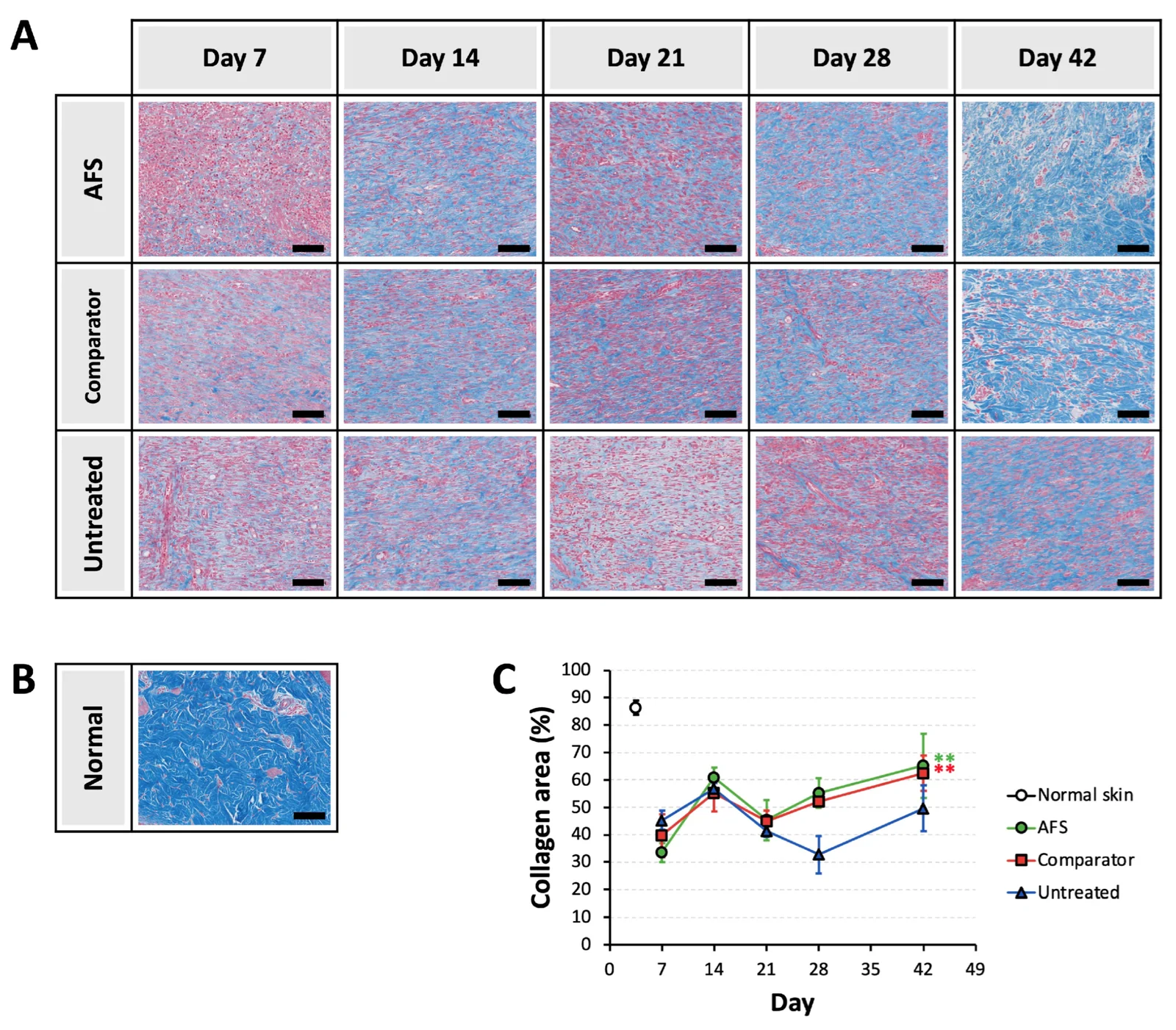
Collagen deposition and dermal matrix remodeling assessed through Masson’s trichrome staining in the porcine full-thickness wound model. (**A**) Representative Masson’s trichrome-stained sections of wounds treated with AFS, comparator (Kerecis Omega3 Wound), or left untreated, on Days 7, 14, 21, 28, and 42 after wounding. Collagen is stained blue and cellular components are stained red. Scale bar = 100 μm. (**B**) Representative Masson’s trichrome-stained section of non-wounded normal skin, shown as a reference. Scale bars: 100 µm (**A**,**B**). (**C**) Quantification of the collagen area, expressed as a percentage of the analyzed wound area. Normal skin was evaluated at a single time point and is shown as a reference value. Data are presented as the mean ± SD. Days 7–28: measurements from multiple sites within a single section per group obtained from one animal (descriptive; no statistical comparison). Day 42: *n* = 4 wounds per group. Colored asterisks indicate significant differences in the correspondingly colored group vs. the untreated group at Day 42 (green, AFS; red, comparator; ** *p* < 0.01). AFS, acellular fish skin; ADM, acellular dermal matrix.

Collagen deposition during wound remodeling was evaluated through Masson’s trichrome (MT) staining, in which collagen fibers are stained blue (Figure 5A,B). The collagen area was quantified as a percentage of the total wound bed area (Figure 5C). Normal skin exhibited an abundant, densely organized collagen network occupying 86.3 ± 2.6% of the dermis (Figure 5B).

Because the Day 7–28 samples were obtained from a single animal, these time points are described without statistical comparison. On Day 7, the collagen area was markedly lower in all wounded groups than in normal skin (AFS, 33.5 ± 3.5%; comparator, 39.7 ± 3.4%; untreated, 42.3 ± 7.9%). The collagen area increased sharply by Day 14 (AFS, 60.9 ± 3.7%; comparator, 55.2 ± 2.0%; untreated, 56.9 ± 6.6%) and decreased transiently at Day 21 (45.4 ± 7.4%, 44.9 ± 2.1%, and 41.4 ± 3.9%, respectively). On Day 28, the collagen area in the untreated group decreased further (32.7 ± 1.4%), whereas the AFS and comparator groups showed higher values (55.2 ± 5.5% and 52.2 ± 6.9%, respectively). On Day 42 (*n* = 4 wounds per group), the collagen area was significantly higher in the AFS and comparator groups (65.2 ± 11.7% and 62.5 ± 8.3%, respectively) than in the untreated group (49.7 ± 6.5%; *p* < 0.01 for both).

Consistent with the quantitative measurements, collagen staining in the AFS group was more extensive and more uniformly distributed throughout the regenerated dermis on Day 42 than at the earlier examined time points (Figure 5A,B). The collagen area in the AFS group on Day 42 corresponded to approximately 76% of the value measured in normal skin. No significant difference in collagen area fraction was detected between the AFS and comparator groups at Day 42.

### 2.6. Type I Collagen Deposition During Dermal Matrix Remodeling

**Figure 6:**
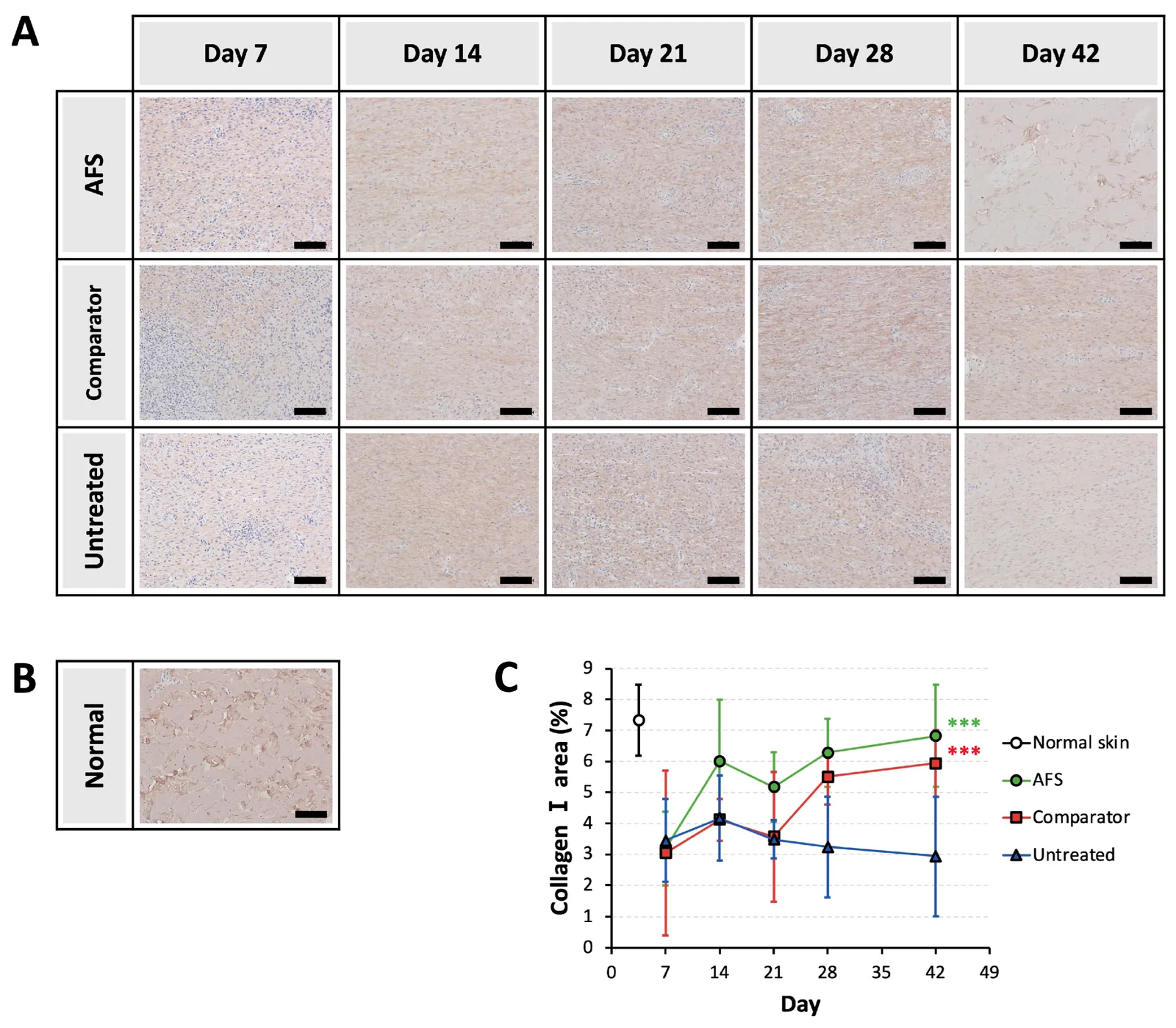
Type I collagen deposition during dermal matrix remodeling assessed by immunohistochemistry in the porcine full-thickness wound model. (**A**) Representative immunohistochemical staining for type I collagen in wounds treated with AFS, comparator (Kerecis Omega3 Wound), or left untreated, on Days 7, 14, 21, 28, and 42 after wounding. Type I collagen areas appear brown, and nuclei are counterstained with hematoxylin (blue). Scale bar = 100 μm. (**B**) Representative type I collagen immunostaining of non-wounded normal skin, shown as a reference. Scale bars: 100 µm (**A**,**B**). (**C**) Quantification of the type I collagen area, expressed as a percentage of the analyzed wound area. Normal skin was evaluated at a single time point and is shown as a reference value. Data are presented as the mean ± SD. Days 7–28: measurements from multiple sites within a single section per group obtained from one animal (descriptive; no statistical comparison). Day 42: *n* = 4 wounds per group. Colored asterisks indicate significant differences in the correspondingly colored group versus the untreated group at Day 42 (green, AFS; red, comparator; *** *p* < 0.001). AFS, acellular fish skin; ADM, acellular dermal matrix.

Type I collagen distribution within the wound bed was evaluated by immunohistochemistry and quantified as the collagen I area fraction (Figure 6). Normal skin showed a collagen I area fraction of 7.3 ± 1.1% (Figure 6B,C).

Because the Day 7–28 samples were obtained from a single animal, these time points are described without statistical comparison. On Day 7, the collagen I areas were 3.2 ± 1.2%, 3.1 ± 1.3%, and 3.5 ± 2.7% in the AFS, comparator, and untreated groups, respectively, less than half of the normal-skin value (Figure 6A).

In the AFS group, the collagen I area increased to 6.0 ± 2.0% on Day 14 and was 5.2 ± 1.1%, 6.3 ± 1.1%, and 6.8 ± 1.7% on Days 21, 28, and 42, respectively. The corresponding values in the comparator group were 4.1 ± 1.4% on Day 14, 5.5 ± 1.6% on Day 28, and 5.9 ± 1.9% on Day 42. In the untreated group, the collagen I-positive area fraction was 4.2 ± 0.7% on Day 14 and decreased to 2.9 ± 1.1% by Day 42.

On Day 28, the collagen I area was higher in the AFS and comparator groups than in the untreated group. On Day 42 (*n* = 4 wounds per group), both the AFS and comparator groups showed significantly higher values than the untreated group (*p* < 0.001 for both comparisons). The Day 42 values in the AFS, comparator, and untreated groups corresponded to approximately 93%, 81%, and 40%, respectively, of the mean value measured in normal skin.

Consistent with the quantitative analysis, collagen I immunostaining in the AFS group on Day 42 appeared dense and broadly distributed throughout the regenerated dermis, whereas the untreated group showed a comparatively sparse and irregular staining pattern (Figure 6A,B). No significant difference in collagen I area was detected between the AFS and comparator groups at Day 42.

## 3. Discussion

In this study, we developed an intact, sheet-form acellular dermal matrix from Japanese amberjack (*Seriola quinqueradiata*) skin and evaluated its physicochemical characteristics, biocompatibility, and wound-healing performance. The resulting amberjack skin-derived ADM (acellular fish skin, AFS) met a commonly applied criterion for tissue decellularization, retained a collagen-rich extracellular matrix (ECM), and exhibited an asymmetric porous architecture, rapid hydration, mechanical integrity under hydrated conditions, and acceptable in vitro and in vivo biocompatibility. In a porcine full-thickness wound model, AFS treatment enhanced early macroscopically estimated re-epithelialization relative to untreated wounds and limited early expansion of the tattooed periwound area. During later remodeling, AFS-treated wounds showed greater total collagen and type I collagen areas than untreated wounds. No significant differences between AFS and the commercial cod skin-derived comparator were detected across the macroscopic, physiological, or histological endpoints. To our knowledge, this is the first preclinical evaluation of an amberjack skin-derived ADM for wound repair and the first head-to-head comparison of a newly developed fish skin ADM with an established commercial fish skin matrix in a porcine full-thickness wound model.

Effective decellularization is a critical prerequisite for xenogeneic ADM development because residual cellular material and nucleic acids may contribute to inflammatory or immunologic responses after implantation [12]. The decellularization and decolorization procedures converted the dark, pigmented raw amberjack skin into a white and pliable sheet while preserving its gross physical continuity. Although this change in appearance is consistent with the removal of pigments and cellular constituents, visual assessment alone does not establish the extent of their removal. More direct evidence of decellularization was provided by the absence of visible nuclei on H&E staining and by the reduction in residual dsDNA to 29.2 ± 7.6 ng/mg dry weight, corresponding to an approximately 99.9% reduction from raw fish skin (RFS) and falling below the commonly applied threshold of 50 ng/mg dry weight for decellularized biologic scaffolds [12]. These results satisfy two of the three commonly cited criteria proposed by Crapo et al. [12]. The third criterion, a residual DNA fragment length of less than 200 bp, was not assessed in this study. In addition, nuclear removal was assessed by H&E staining alone, without nuclear-specific staining such as DAPI. Therefore, although the present data indicate effective removal of nuclear material, the extent of decellularization should be interpreted with these limitations in mind.

ATR-FTIR analysis further showed close correspondence between the collagen-associated amide bands in RFS and AFS. The preservation of the amide A, I, II, and III band positions and overall spectral profiles suggests that the principal molecular structure of collagen was largely maintained during processing, consistent with previous observations in fish-derived decellularized matrices [18,37,38]. In contrast, the relative change in the C–H stretching region between 2800 and 3000 cm^−1^ may reflect the removal or alteration of aliphatic constituents, including cell membrane- and lipid-associated components, during decellularization and washing [12,13,14,38]. Although ATR-FTIR analysis provided information on collagen-associated functional groups, it does not directly establish the preservation of the native collagen triple-helical structure or determine its thermal denaturation temperature. Differential scanning calorimetry (DSC) was not performed in the present study; therefore, the thermal stability of AFS collagen remains to be characterized. Further studies using DSC are warranted to determine the thermal denaturation temperature and provide complementary evidence regarding collagen stability following decellularization and processing. Collagen was the predominant component of the final AFS, while measurable quantities of sGAGs and elastin were also detected. Because the hydroxyproline content of fish collagen is generally lower than that of mammalian collagen, the conversion factor used here may underestimate the collagen content of AFS. The absolute collagen content should therefore be regarded as an approximation. The presence of these non-collagenous components indicates that the decellularization processing did not completely eliminate the biochemical diversity of the matrix. Such components can contribute to ECM hydration, molecular interactions, and cellular responses, although their biological activity was not directly examined in this study [12,13,14]. In addition, the lipid and fatty acid composition of AFS was not analyzed. It therefore remains unknown whether the omega-3 fatty acids reported in other fish skin grafts [22,23] are retained in AFS, and no contribution of such lipids to the observed wound-healing response can be inferred from the present data.

The structural features of the AFS may contribute to its performance as a wound matrix. SEM demonstrated a pronounced asymmetric microarchitecture consisting of a porous dermal-side surface, a stratified cross-section, and a denser scale-side surface, with a bulk porosity of 29.1 ± 8.0% determined by ethanol displacement. Similar structural heterogeneity has been reported in other fish skin-derived matrices and likely reflects the intrinsic multilayered organization of native fish skin [17,18,37]. From a wound care perspective, the open wound-facing surface may facilitate fluid exchange and cellular infiltration, whereas the denser outer surface may provide structural support and barrier-like protection. Consistent with this morphology, AFS absorbed saline rapidly and reached an apparent swelling equilibrium within 1 min. Although saline swelling does not fully reproduce the complexity of wound exudate, this rapid fluid-uptake behavior suggests that the material can interact promptly with a moist wound environment after application.

The bulk porosity of AFS (29.1 ± 8.0%) is lower than values reported for freeze-dried collagen sponges and for other fish skin-derived acellular matrices (approximately 80–90%) [18,28]. This difference likely reflects both the material and the measurement. As an intact native dermis, AFS includes the dense scale-side layer across its full thickness, whereas SEM showed large, interconnected pores on the dermal-side surface. In addition, the use of a geometric volume and surface blotting in a thin sheet may underestimate porosity, and measurements in the dry state may not represent the pore structure of the hydrated material as applied. Consistent with this, implanted AFS supported host cell infiltration, neovascularization, and progressive tissue integration (Figure 2I), and was no longer macroscopically distinguishable in porcine wounds after the first week (Figure 3A). Nevertheless, because pore size distribution, interconnectivity, and infiltration depth were not quantified, whether the low bulk porosity limits cellular ingrowth into denser regions of the matrix warrants further investigation.

Under hydrated conditions, AFS exhibited a characteristic nonlinear J-shaped stress–strain response, with an initial low-stiffness toe region followed by progressive strain stiffening until failure. This behavior is characteristic of collagen-rich soft tissues and is generally attributed to the progressive recruitment, straightening, and alignment of initially crimped or loosely organized collagen fibers [39,40]. The hydrated AFS showed a tensile strength of 2.4 ± 0.4 MPa, an elongation at break of 59.6 ± 11.3%, and an apparent initial Young’s modulus of 0.8 ± 0.3 MPa calculated over the 0–10% strain range. These results indicate that the material combined a compliant low-strain response with tensile integrity and substantial extensibility. The initial Young’s modulus of 0.8 ± 0.3 MPa falls within the broad range reported for native skin (approximately 10 kPa to 50 MPa) [40,41,42] and is comparable to values reported for collagen membranes (~0.8–1.0 MPa) [43] and freeze-dried porous collagen scaffolds (~0.89 MPa) [44] intended for wound-healing applications. Because a lower modulus combined with greater extensibility has been associated with increased elasticity and conformability of hydrated collagen dressings to irregular wound beds [45], this compliance may be advantageous for one-step coverage of irregular wound surfaces rather than representative of a mechanical limitation. However, direct comparisons among studies are limited by differences in hydration state, specimen orientation and geometry, strain rate, and testing protocol.

Biocompatibility is a prerequisite for any xenogeneic wound-covering material to advance toward clinical use, and the present findings provide converging in vitro and in vivo evidence in support of AFS in this respect. In an extract-based cytotoxicity assay, L929 fibroblasts exposed to AFS extract retained a normal, well-spread morphology and a viability of 80.3 ± 4.3% relative to the control, above the 70% threshold defined in ISO 10993-5, with no indication of cytotoxic leachables. Given the effective decellularization described above, this result is consistent with the removal of residual detergent, nucleic acid fragments, and other processing byproducts that are among the more common sources of cytotoxicity in decellularized biologic scaffolds. Eight-week subcutaneous implantation in rats provided further support: no gross evidence of abscess formation, necrosis, or extrusion was observed at any time point. The local tissue response progressed from a self-limited inflammatory infiltrate at week 1 to neovascularization and host-derived fibrous tissue deposition, while the implant became progressively less distinct. This pattern of early, non-destructive inflammation followed by vascularized tissue ingrowth is expected for a biologically derived, collagen-rich scaffold and argues against a sustained foreign-body or chronic inflammatory reaction to AFS.

Wounds treated with AFS or the comparator showed faster re-epithelialization than untreated wounds during the first one to two weeks. From Day 14 onward, however, re-epithelialization values converged across all three groups, and by Day 42 essentially all wounds, including untreated controls, had closed. This convergence likely reflects the substantial intrinsic healing capacity of full-thickness wounds in young, otherwise healthy pigs, and suggests that the main benefit of AFS application in this model was to shorten the early lag phase of epithelial coverage rather than to change the overall healing endpoint. Whether this early advantage would persist, or become more pronounced, in a compromised or chronic wound setting where endogenous re-epithelialization is impaired remains an open question.

A related early effect was seen in periwound skin contraction. Untreated wounds showed an approximately 18% increase in the normalized tattooed area by Day 7, consistent with acute wound-margin retraction, whereas AFS- and comparator-treated wounds stayed close to baseline over the same period. Limiting this early contraction could be clinically meaningful, since exaggerated early retraction of the wound margin has been linked to greater scar tension and less favorable cosmetic outcomes in full-thickness wounds. As with re-epithelialization, this difference did not persist once granulation and remodeling were underway. By Day 42, the periwound area had decreased to a similar extent in all three groups. This late convergence likely reflects the natural course of scar maturation rather than a treatment-specific effect.

Several mechanisms may account for these early effects. First, the collagen-rich ECM sheet may act as a provisional scaffold that covers the exposed wound bed and provides a substrate for keratinocyte and fibroblast migration, as proposed for other fish skin-derived matrices [17,18,19]. Second, the adherent and conformable matrix may have mechanically limited early wound-margin retraction. Because re-epithelialization was calculated from the reduction in open wound area, the early retraction observed in untreated wounds would enlarge their open area and thereby contribute to the difference in re-epithelialization on Day 7. Third, the hydrated matrix may help maintain a moist wound interface that favors epithelial migration [46], although the foam secondary dressing applied only to the matrix-treated wounds may also have contributed. Fourth, the mild host response to AFS, without a foreign-body reaction, may have allowed early repair to proceed without prolonged inflammation. Non-collagenous ECM components such as sGAGs may also influence cell behavior [12,13,14], but this was not examined. Notably, these early effects were similar for AFS and the comparator. This suggests that they reflect properties shared by fish skin-derived ECM matrices, or the treatment protocol, rather than features specific to amberjack skin. Because the relevant biological markers were not assessed, these mechanisms remain hypotheses.

The recovery of skin barrier function, assessed through TEWL and stratum corneum hydration, followed a broadly similar course in all three groups, with values elevated relative to normal skin for the first two to three weeks and normalizing thereafter. The absence of significant differences among the AFS, comparator, and untreated groups at any time point indicates that AFS neither accelerated nor delayed the physiological restoration of the epidermal barrier relative to the other conditions. Because barrier recovery depends primarily on the underlying keratinocyte differentiation program rather than on the properties of a temporary wound covering, this outcome is not unexpected, but it does indicate that AFS application did not interfere with normal barrier restoration.

Histological findings were consistent with these functional observations. Because the Day 7–28 samples were obtained from a single animal, the time-course observations are descriptive. In that animal, inflammatory cell infiltration in AFS-treated wounds appeared to decrease from severe at Day 7, and by Day 42 (*n* = 4 wounds per group) it was mild in all three groups. No foreign-body granulomatous reaction was observed at any time point. Granulation tissue formation and angiogenesis appeared similar among the groups during Days 7–28, and none of the histologically scored parameters differed significantly between groups at Day 42.

Beyond these general indices of remodeling, quantitative collagen staining showed that the total collagen-stained area and the collagen I-immunopositive area were significantly higher in AFS-treated wounds than in untreated wounds at Day 42 (*n* = 4 wounds per group), reaching approximately 76% and 93% of the corresponding normal skin values, respectively. These findings indicate greater collagen deposition within the wound bed of AFS-treated wounds. However, because Masson’s trichrome staining and area-based immunohistochemical quantification reflect the extent and distribution of collagen rather than its maturity, fiber organization, or crosslinking, the present data do not establish that AFS promotes a more mature or better-organized dermal matrix. Such conclusions would require additional analyses, such as picrosirius red staining under polarized light or second-harmonic generation imaging. In addition, neither of these methods distinguishes host-derived from scaffold-derived collagen, so a contribution of residual fish collagen to these measurements cannot be fully excluded. Notably, no significant differences between AFS and the comparator were detected at any examined time point across the macroscopic, physiological, and histological endpoints, including these collagen measures, although this study was not designed to demonstrate equivalence.

Taken together with the physicochemical and biocompatibility characterization, these results suggest that amberjack skin is a promising candidate source of fish skin-derived ADM, in addition to the more extensively studied cod and tilapia. Differences among fish species in ECM composition, such as collagen subtype distribution, native crosslink density, collagen thermal stability, or the relative content of non-collagenous components such as sGAGs and elastin, could plausibly affect degradation kinetics or the local host response. However, because these properties were not compared directly between AFS and the cod skin-derived comparator in this study, such species-dependent differences remain hypotheses that warrant side-by-side characterization in future studies. From a supply standpoint, amberjack aquaculture in Northeast Asia generates substantial quantities of skin as a low-value processing byproduct, and these results suggest that this material can be converted into a wound-covering ADM that showed no significant differences from an existing commercial product in this model, potentially offering a regionally sourced alternative that could help diversify the raw-material base for this class of biomaterials.

Recent advances in wound dressing development have increasingly focused on integrating structural and functional properties to address the complex requirements of wound management. For example, kaolin/chitin composite aerogels have been investigated for their porous architecture, water absorption and retention, and hemostatic performance [47]. Recent work on chitosan and chitosan nanofibers has also highlighted the potential of functional modification to introduce properties such as antimicrobial activity and hemostasis, while broadening their applications in wound care [48]. In addition, a chitosan/poly (vinyl alcohol)-based biosponge incorporating ciprofloxacin, metformin, and calcium peroxide has been developed to combine antibacterial and anti-inflammatory activities with sustained oxygen generation for diabetic wound treatment [49]. These studies illustrate the growing emphasis on multifunctional wound dressings that combine fluid management with additional therapeutic functions. In comparison, the present study investigated a fish skin-derived extracellular matrix scaffold, focusing on its physicochemical characteristics, biocompatibility, and wound-healing performance in a porcine full-thickness wound model. The asymmetric architecture and rapid hydration of AFS may be relevant to its use as a wound-covering matrix; however, antimicrobial activity, hemostatic efficacy, and active-agent delivery were not specifically evaluated in the present study. Further research would be required to determine whether these or other functional properties could be incorporated into, or combined with, AFS-based wound management strategies.

Several limitations should be considered in interpreting these results. Histological analysis from Day 7 through Day 28 relied on tissue from a single designated animal per time point, which limits statistical power at these earlier stages and means the corresponding findings should be regarded as descriptive rather than confirmatory; only the Day 42 histology included the full complement of four wounds per group. The wound-healing assessments were also based on four wounds per group obtained from only two animals, and each wound was analyzed as an independent observation. Because wounds within the same animal are not fully independent, the reported *p*-values may overstate the strength of evidence, and the statistical comparisons in the porcine model should be regarded as exploratory. The porcine model is widely used because of its structural similarity to human skin. However, this study used healthy young animals with acute, surgically created wounds. Such wounds heal more readily and predictably than the chronic, comorbidity-associated wounds for which fish skin-derived ADMs are often used clinically, such as diabetic foot ulcers and venous leg ulcers. The early advantage of AFS in re-epithelialization and periwound contraction may therefore differ in magnitude under impaired-healing conditions. In addition, the untreated wounds received gauze only, whereas the AFS- and comparator-treated wounds were also covered with a foam dressing; the differences from the untreated group may therefore partly reflect the secondary dressing rather than the matrices alone, whereas the comparison between AFS and the comparator is not affected. The 42-day observation period also does not capture the full degradation timeline of the matrix or longer-term outcomes such as final scar quality and the mechanical properties of the regenerated tissue. In addition, the cross-reactivity of the anti-collagen type I antibody with amberjack- or cod-derived collagen was not evaluated, and neither MT staining nor IHC distinguishes newly synthesized porcine collagen from residual scaffold-derived collagen. Although the matrices were no longer macroscopically distinguishable after the first week and the differences in collagen area emerged only in the later remodeling phase, species-specific antibodies or matrix-only staining controls would be needed to confirm the host origin of the deposited collagen. Furthermore, the present study relied on routine histology and collagen staining and did not assess inflammatory or regenerative biomarkers, such as macrophage phenotype markers (e.g., CD68 and CD206), endothelial markers (e.g., CD31), proliferation markers (e.g., Ki-67), or cytokine and growth factor expression; the mechanisms underlying the observed healing response therefore remain to be elucidated. Finally, this study characterized a single production batch of AFS, and batch-to-batch or seasonal variability in raw amberjack skin, which could influence ECM composition and mechanical behavior, was not assessed and warrants further investigation before clinical translation.

## 4. Materials and Methods

### 4.1. Preparation of the Amberjack Skin-Derived ADM

Amberjack skin was purchased from Samsam Haemul Fisheries Association Corporation (Geoje-si, Gyeongsangnam-do, Republic of Korea). Briefly, the skins were thawed and trimmed to remove scales, subcutaneous fat, and residual muscle tissue. The trimmed raw fish skins were immersed in an aqueous solution of 0.5% (*w*/*v*) sodium dodecyl sulfate (SDS; Sigma-Aldrich, St. Louis, MO, USA) under agitation for at least 12 h for decellularization. The decellularized skins were then thoroughly washed with distilled water to remove residual detergent. Subsequently, decolorization was performed by immersing the decellularized skins in an aqueous bleaching solution containing 10% (*w*/*v*) ammonium persulfate (Daejung Chemicals & Metals, Siheung, Republic of Korea), 2.1% (*w*/*v*) sodium metasilicate (Sigma-Aldrich), and 5% (*v*/*v*) hydrogen peroxide (Samchun Pure Chemical, Pyeongtaek, Republic of Korea) under agitation for 1.5 h. After decolorization, the skins were washed again with distilled water until residual bleaching agents were removed. The washed skins were frozen at ≤−20 °C for at least 6 h and then freeze-dried for at least 48 h using a freeze dryer (MP-90100, Mareuda Co. Ltd., Gwangju, Republic of Korea), with the freeze-drying temperature starting at ≤−20 °C and ending at ≥15 °C. The overall preparation process is illustrated in Figure 1A. The resulting AFS sheets were packaged in Tyvek^®^ pouches and sterilized with ethylene oxide (EO) gas (Green Sterilization Co., Ltd., Gunpo-si, Republic of Korea) using a validated cycle in accordance with ISO 11135 [50]. The cycle consisted of preconditioning at 10–45 °C for at least 120 min, air removal to −0.85 kgf/cm^2^, humidification to 50 ± 25% relative humidity (RH), and EO injection to 0.7 ± 0.05 kgf/cm^2^ at an EO concentration of 700–800 mg/L. EO exposure was performed at 40 ± 5 °C and 50 ± 25% RH for 240 min, followed by at least seven flushing cycles. The sterilized AFS was then aerated at 10–40 °C for 48 h in a dedicated aeration room. Residual EO and ethylene chlorohydrin (ECH) were determined in accordance with ISO 10993-7 [51], and neither was detected in the AFS extract (limits of detection: 0.641 and 1.735 µg/mL, respectively). The appearance of the final AFS sheet is shown in Figure 1B. The removal of residual processing chemicals was confirmed by elemental analysis of marker elements absent from the raw material: sulfur, derived from SDS and ammonium persulfate, was not detected by X-ray photoelectron spectroscopy (XPS) in aqueous extracts of the final AFS (6 cm^2^/mL, 37 °C, 72 h), and silicon, derived from sodium metasilicate, was not detected by inductively coupled plasma optical emission spectroscopy (ICP-OES) in acid-digested AFS (*n* = 3 each).

### 4.2. Histological Evaluation of Decellularization

To histologically confirm decellularization in the final AFS, hematoxylin and eosin (H&E) staining was performed. The AFS was fixed in 4% paraformaldehyde (Sigma-Aldrich), dehydrated through a graded sucrose (Sigma-Aldrich) solution series, embedded in optimal cutting temperature (OCT) compound (Tissue-Tek^®^, Sakura Finetek, Torrance, CA, USA), and cryosectioned at a thickness of 5 µm using a cryostat (Leica CM3050 S, Leica Biosystems, Nussloch, Germany). The sections were stained with hematoxylin (Sigma-Aldrich) and eosin (Sigma-Aldrich) according to a standard histological protocol and observed under a brightfield microscope (Eclipse Ti, Nikon, Tokyo, Japan). Decellularization was qualitatively evaluated by confirming the absence of visible nuclei in the final AFS matrix.

### 4.3. Residual DNA Quantification

To quantitatively assess the efficiency of decellularization, residual double-stranded DNA (dsDNA) content was measured in both non-decellularized raw fish skin (RFS) and AFS. Lyophilized samples (*n* = 3 per group) were weighed and enzymatically digested in Tris-EDTA (TE) buffer containing Proteinase K (10 mg/mL; Bioneer, Daejeon, Republic of Korea) until the tissue was completely solubilized. The resulting sample lysates were directly subjected to dsDNA quantification. The dsDNA content was quantified using the Quant-iT™ PicoGreen™ dsDNA Assay Kit (Thermo Fisher Scientific, Waltham, MA, USA) according to the manufacturer’s instructions. Briefly, aliquots of the sample lysates were diluted in TE buffer and mixed with the PicoGreen working solution. Fluorescence intensity (excitation/emission: 480 nm/520 nm) was measured using a microplate reader (Varioskan LUX, Thermo Fisher Scientific). Residual DNA content was calculated from a standard curve and expressed as nanograms of dsDNA per milligram of dry tissue weight (ng/mg).

### 4.4. Attenuated Total Reflectance–Fourier Transform Infrared Spectroscopy (ATR-FTIR)

To evaluate the preservation of the ECM chemical structure after decellularization, ATR-FTIR spectroscopy was performed on RFS and AFS using an ATR-FTIR spectrometer (Cary 670, Agilent Technologies, Santa Clara, CA, USA). Each lyophilized sample was placed directly on the ATR crystal, and spectra were collected over a wavenumber range of 4000–400 cm^−1^ at a resolution of 4 cm^−1^, with 32 scans per spectrum.

### 4.5. Quantification of ECM Composition

The biochemical composition of the final AFS was evaluated by quantifying collagen, sulfated glycosaminoglycan (sGAG), and elastin content. Lyophilized AFS samples were weighed, and the contents of collagen, sGAG, and elastin were quantified using a Hydroxyproline Assay Kit (Sigma-Aldrich), Blyscan™ Sulfated Glycosaminoglycan Assay Kit (Biocolor Ltd., Carrickfergus, UK), and Fastin™ Elastin Assay Kit (Biocolor Ltd.), respectively, according to the manufacturers’ instructions (*n* = 10 per component). For collagen quantification, lyophilized AFS samples (10 mg) were hydrolyzed in 12 M HCl at 120 °C for 3 h, and the hydroxyproline content of the hydrolysates was determined using a Hydroxyproline Assay Kit (MAK569, Sigma-Aldrich) according to the manufacturer’s instructions. Collagen content was calculated by multiplying the hydroxyproline content by a conversion factor of 7.46 [52]. Absorbance was measured using a microplate reader (Varioskan LUX, Thermo Fisher Scientific). The results were normalized to the dry weight of each sample and expressed as µg/mg dry weight.

### 4.6. Scanning Electron Microscopy (SEM)

The surface morphology of the AFS was examined through scanning electron microscopy (SEM). AFS samples were prepared to expose the outer surface (scale-side surface), cross-section, and inner surface (dermal-side surface). The samples were sputter-coated with platinum and observed using SEM (Inspect F50, FEI, Hillsboro, OR, USA) at an accelerating voltage of 10 kV.

### 4.7. Porosity

The porosity of the AFS was determined using an ethanol displacement method [18,28]. Briefly, lyophilized AFS samples (*n* = 3) were weighed to obtain the initial dry weight (W_0_). Each sample was then immersed in absolute ethanol (Daejung Chemicals & Metals) and placed in a desiccator (IN-BG1KS-AD, iNexus Inc., Seongnam, Republic of Korea) under reduced pressure at room temperature for 15 min to allow complete infiltration into the pore spaces. After removal from ethanol, excess ethanol on the sample surface was gently blotted with filter paper, and the sample was immediately reweighed (W_1_). The geometric volume of each sample (V_S_) was calculated from its dimensions. Porosity was calculated using the following equation:

(1) $$\mathrm{Porosity}\;\left(\%\right)=\;\frac{W_{1}-\;W_{0}}{\rho V_{s}}\;\times \;100,$$

where *ρ* is the density of ethanol (0.789 g/mL).

### 4.8. Swelling Ratio

Because AFS is fully rehydrated in normal saline before application, its swelling behavior was evaluated to characterize its fluid-uptake kinetics and hydrated-state stability and to confirm the hydration time required before use. The swelling behavior of the AFS was evaluated using a gravimetric method in triplicate (*n* = 3). AFS samples (10 cm × 7 cm) were weighed to obtain the initial dry weight (W_0_), after which each sample was immersed in normal saline (0.9% NaCl). At predetermined time points (5, 10, and 30 s and 1, 2, 5, and 10 min), the sample was removed from the normal saline, gently blotted to remove excess surface liquid, and immediately weighed to obtain the swollen weight (W_t_). The swelling ratio was calculated based on the increase in sample weight relative to the initial dry weight using the following equation:

(2) $$\mathrm{Swelling}\;\mathrm{ratio}\;\left(\%\right)\;=\;\frac{W_{t}\;-\;W_{0}}{W_{0}}\;\times \;100.$$

### 4.9. Tensile Properties

The tensile properties of the AFS were evaluated using a universal testing machine (UTM; Instron 5942, Instron, Norwood, MA, USA). AFS specimens (*n* = 3) were cut to a width of 6 mm and a gauge length of 20 mm, with a thickness of approximately 0.4 mm. Prior to testing, the specimens were hydrated in normal saline for at least 1 min to ensure that testing was performed in the fully swollen state corresponding to clinical application. Each hydrated specimen was mounted on the UTM and stretched at a crosshead speed of 20 mm/min. The maximum tensile load was recorded. Tensile strength was defined as the maximum engineering stress, and elongation at break as the engineering strain at failure. Young’s modulus was determined as the slope of a linear regression fitted to the 0–10% strain region of the stress–strain curve.

### 4.10. In Vitro Cytotoxicity Evaluation

In vitro cytotoxicity of the AFS was evaluated using an extraction method in accordance with ISO 10993-5. L929 mouse fibroblasts (ATCC, Manassas, VA, USA) were cultured in Minimum Essential Medium (MEM; Gibco, Grand Island, NY, USA) supplemented with 10% fetal bovine serum (FBS; Gibco) and 1% penicillin–streptomycin (Welgene, Daegu, Republic of Korea). The cells were seeded into 6-well plates at a density of 2 × 10^5^ cells/well in 2 mL of culture medium and allowed to attach for 24 h at 37 °C in a humidified 5% CO_2_ incubator (Forma 371, Thermo Fisher Scientific, Waltham, MA, USA) prior to extract exposure.

The AFS extract was prepared in accordance with ISO 10993-12 [53]. Sterilized AFS was immersed in culture medium at a surface area-to-medium ratio of 6 cm^2^/mL and incubated for 24 h at 37 °C. The resulting extract was used as the test article without further dilution. Culture medium incubated under identical conditions without AFS served as the control.

After the 24 h attachment period, the culture medium in each well was replaced with 2 mL of the AFS extract or control medium, and the cells were incubated for an additional 24 h at 37 °C in 5% CO_2_. After extract exposure, cell morphology was observed in the control and AFS extract-treated groups using an inverted microscope (TS100F, Nikon, Tokyo, Japan). For quantitative analysis, 1 mL of MTT solution (Invitrogen, Thermo Fisher Scientific, Waltham, MA, USA) was added to each well and incubated for 2 h at 37 °C. The resulting formazan crystals were dissolved in DMSO (Sigma-Aldrich), and absorbance was measured at 540 nm using a microplate reader (Varioskan LUX, Thermo Fisher Scientific). Cell viability was calculated as the percentage of absorbance in the AFS extract-treated group relative to that of the control group. All experiments were performed in triplicate (*n* = 3).

### 4.11. In Vivo Subcutaneous Implantation in Rats

The in vivo biocompatibility and biodegradation of AFS samples were evaluated through subcutaneous implantation in Sprague–Dawley (SD) rats. All animal procedures were approved by the Institutional Animal Care and Use Committee (IACUC) of HLBbioStep Co., Ltd. (approval number: S25-RP-0454) and were performed in compliance with national guidelines for the care and use of laboratory animals.

Twelve male SD rats (9 weeks old, 288.9 ± 5.5 g) were obtained from KOATECH (Pyeongtaek, Republic of Korea) and then acclimatized for 7 days prior to the experiment. The AFS samples were cut into 1 cm × 1 cm pieces and hydrated in sterile normal saline (0.9% NaCl) for at least 1 min. The rats were anesthetized through intraperitoneal injection of a mixture of tiletamine-zolazepam (Zoletil^®^, Virbac, Carros, France) and xylazine (Rompun^®^, Bayer, Leverkusen, Germany). The dorsal region was shaved and disinfected with 70% ethanol. An approximately 2 cm skin incision was made on the left lateral dorsal region, and a subcutaneous pocket was created by blunt dissection. One hydrated AFS sample was implanted into the subcutaneous pocket and positioned flat without folding. Another hydrated AFS sample was implanted subcutaneously in the right lateral dorsal region using the same procedure. The skin incisions were closed with 4-0 polypropylene sutures (Prolene^®^, Ethicon, Johnson & Johnson, Somerville, NJ, USA), and the surgical sites were disinfected with povidone–iodine. The implantation sites were marked on the skin using a surgical skin marker.

Body weights were measured on the day of implantation, once weekly, and on the day of euthanasia. At predetermined time points (1, 3, 5, and 8 weeks after implantation), three rats were euthanized at each time point, and a veterinarian performed a gross evaluation of the implantation sites (*n* = 6 per time point) for the presence of inflammation, encapsulation, hemorrhage, necrosis, discoloration, and residual AFS material in accordance with ISO 10993-6. After gross evaluation, the implantation sites, including the remaining AFS material and surrounding tissues, were excised and fixed in 10% neutral-buffered formalin. The tissue samples were then processed for routine histopathological examination, embedded in paraffin, sectioned, and stained with hematoxylin and eosin (H&E). A pathologist performed microscopic evaluation of the tissue samples, including assessment of residual AFS material, inflammatory cell infiltration, necrosis, neovascularization, fibrosis, and fatty changes, in accordance with ISO 10993-6. Histological sections were observed under a light microscope (BX53, Olympus, Tokyo, Japan) at magnifications of 12.5× and 400×.

### 4.12. In Vivo Full-Thickness Wound Model in Pigs

The in vivo wound-healing efficacy and safety of AFS were evaluated in a porcine full-thickness wound model. This study was conducted at HLBbioStep Co., Ltd. (Incheon, Republic of Korea), a specialized preclinical testing institute, under veterinary supervision. The study protocol was approved by the IACUC of HLBbioStep Co., Ltd. (approval number: KNOTUS IACUC 22-KE-0289), and all animal procedures were performed in compliance with national guidelines for the care and use of laboratory animals.

#### 4.12.1. Wound Creation and Treatment

Three male domestic pigs (35.3 ± 1.4 kg) were obtained from XPBio (Anseong, Republic of Korea) and acclimatized for 11 days prior to the experiment, and only healthy animals were used in the study. The pigs were premedicated with an intramuscular injection of tiletamine-zolazepam (Zoletil^®^, Virbac, Carros, France) and xylazine (Rompun^®^, Bayer, Leverkusen, Germany). General anesthesia was maintained with isoflurane (1–3% in oxygen) delivered via endotracheal intubation. The pigs were placed in the prone position, and the dorsal skin was disinfected. Six wound sites were marked on the dorsum of each animal as 4 cm × 4 cm squares, and a 5 cm × 5 cm tattoo was made around each wound site to assess skin contraction during wound healing. Additional non-wounded control tattoos were placed on the dorsum of two animals (pig #2 and pig #3) to correct changes in skin surface area associated with animal growth. Full-thickness wounds were created by excising the marked skin area with a scalpel, including the entire epidermis and dermis, down to the subcutaneous adipose tissue, which was preserved. Because the dermal–subcutaneous interface provides a consistent anatomical landmark, wound depth was standardized anatomically rather than by a fixed depth. All wounds were created by the same veterinary surgeon, and the wound sites were placed symmetrically on both sides of the dorsal midline at comparable craniocaudal levels. The wounds and surrounding tattoos were photographed with a flexible ruler placed against the skin surface. The excised full-thickness skin was retained for histopathological evaluation as normal skin tissue.

Each pig received six full-thickness wounds, and three treatment groups were randomly assigned, with two wounds per group: (1) untreated control, (2) comparator (Kerecis Omega3 Wound; Kerecis, Isafjordur, Iceland), and (3) AFS. The untreated control wounds received no test material. The comparator and AFS samples were hydrated in sterile normal saline (0.9% NaCl) for at least 1 min before application to the wound sites. Both materials were applied once on the day of wound creation (Day 0) and maintained for up to 6 weeks. The wounds treated with the comparator and AFS were covered with a non-adhesive hydrophilic foam dressing (Medifoam; Genewel, Seongnam, Republic of Korea) as a secondary dressing to maintain hydration of the matrices, whereas the untreated control wounds received no foam dressing. All wound sites were then covered with dry gauze dressings. Dressings were changed twice weekly during weeks 0–1 and once weekly from week 2 onward. No systemic or topical antibiotics were administered during the study. Wounds were inspected for clinical signs of infection, including purulent discharge, abnormal odor, spreading erythema, and swelling, at each dressing change, and no wound infection was observed in any wound throughout the study.

#### 4.12.2. Study Design and Animal Allocation

One of the three pigs (pig #1) was designated exclusively for serial histopathological sampling on Days 7, 14, 21, and 28 and was euthanized on Day 28 after the final tissue collection. The remaining two pigs (pig #2 and pig #3) were used for wound-healing assessments, including wound area measurement, TEWL, and skin hydration. On Day 42, after completion of all wound-healing assessments, pig #2 and pig #3 were euthanized, and tissue samples were collected from all wound sites for histopathological evaluation.

#### 4.12.3. Wound Area and Skin Contraction

Wound healing was quantitatively assessed by measuring re-epithelialization and skin contraction. In pig #2 and pig #3, photographs of each wound and the surrounding tattoo were obtained with a flexible ruler included in each image on Days 4, 7, 11, 14, 21, 28, 35, and 42 after treatment. The open wound area, wound tattoo area, and control tattoo area were measured from the photographs using ImageJ software (version 1.53, National Institutes of Health, Bethesda, MD, USA). The percentage of re-epithelialization was calculated as the reduction in open wound area relative to the initial wound area, as follows:

(3) $$\%\;\mathrm{Re-epithelialization}\;=\;\frac{{\mathrm{OWA}}_{D_{0}}-\;{\mathrm{OWA}}_{D_{x}\;}}{{\mathrm{OWA}}_{D_{0}}}\;\times \;100,$$

where ${\mathrm{OWA}}_{D_{0}}$ and ${\mathrm{OWA}}_{D_{x}}$ represent the open wound area on Day 0 and Day x, respectively.

To evaluate skin contraction, the wound tattoo area at each time point was first normalized using the change in the control tattoo area to correct for growth-related changes in skin surface area:

(4) $$\mathrm{Normalized}\;{\mathrm{WTA}}_{D_{x}}=\;{\mathrm{WTA}}_{D_{x}}\times \;\frac{{\mathrm{CTA}}_{D_{0}}}{{\mathrm{CTA}}_{D_{x}}}.$$

The percentage of skin contraction was then calculated as follows:

(5) $$\mathrm{Normalized}\;\mathrm{wound}\;\mathrm{tattoo}\;\mathrm{area}\;\left(\%\;\mathrm{of}\;\mathrm{Day}\;0\right)=\;\frac{\mathrm{Normalized}\;{\mathrm{WTA}}_{D_{x}}}{{\mathrm{WTA}}_{D_{0}}}\;\times \;100,\;$$

where *WTA* and *CTA* represent the wound tattoo area and control tattoo area on Day 0 and Day x, respectively.

#### 4.12.4. Transepidermal Water Loss (TEWL) and Skin Hydration

To evaluate the recovery of skin barrier function during wound healing, TEWL and skin hydration were measured at each wound site using Tewameter^®^ TM 300 (Courage+Khazaka electronic GmbH, Köln, Germany) and Corneometer^®^ CM 825 probes (Courage+Khazaka electronic GmbH), respectively, connected to a Cutometer^®^ Dual MPA 580 system (Courage+Khazaka electronic GmbH). Measurements were performed on Days 7, 14, 21, and 42 after treatment. At each wound site, TEWL and skin hydration were measured separately at the wound center and wound edge. TEWL was recorded for approximately 12 s until a stable plateau was reached, and three consecutive measurements were obtained at each measurement location. The mean of the three measurements was used for analysis. For skin hydration, three consecutive readings were obtained at each measurement location, and the mean value was recorded. Measurements from non-wounded normal skin served as the baseline reference.

#### 4.12.5. Histopathological Evaluation

Histopathological evaluation was performed to assess wound-healing progression and to confirm the safety of the applied materials with respect to inflammatory responses. Tissue samples were collected on Days 7, 14, 21, 28, and 42 after treatment. For pig #1, tissue samples were collected as full-thickness strips, approximately 0.5 cm in width and 5–6 cm in length, and oriented to include the wound-healing boundary and the full extent of the surrounding tattoo. At each time point, one strip was collected from each treatment group (*n* = 1 per group per time point). Within each treatment group, sampling was alternated between the two wound sites at successive time points to avoid sampling the same wound at consecutive time points. For pig #2 and pig #3, tissue samples were collected from all wound sites on Day 42 using the same procedure (*n* = 4 per group).

All tissue samples were fixed in 10% neutral-buffered formalin, dehydrated through a graded ethanol series, cleared in xylene, embedded in paraffin, and sectioned. Sections were stained with H&E for histological evaluation and with Masson’s trichrome (MT) to assess the collagen-stained area and its distribution. Immunohistochemical staining for collagen type I was performed using a mouse monoclonal anti-collagen type I antibody (COL-1; ab6308, Abcam, Cambridge, UK) according to a standard paraffin-section IHC protocol. Cross-reactivity of this antibody with fish-derived collagen was not evaluated. The stained sections were examined under a light microscope to evaluate wound tissue regeneration.

For H&E-stained sections, histopathological changes were evaluated quantitatively using a pathology scoring system, as shown in Table 1 [54]. For MT-stained sections, the collagen-stained area was measured and expressed as both the absolute area (µm^2^) and percentage of the total tissue area. For IHC-stained sections, collagen type I expression was quantified by measuring the positively stained area (µm^2^) and percentage of the total tissue area. Excised normal skin obtained at the time of wound creation served as the normal tissue reference for MT and IHC analyses.

### 4.13. Statistical Analysis

All quantitative data are expressed as means ± standard deviation (SD), and the number of replicates (*n*) for each measurement is indicated in the corresponding figure legend, table, or relevant Materials and Methods subsection. Statistical analyses were performed using Prism 7.04 (GraphPad Software Inc., San Diego, CA, USA).

For comparisons between two independent groups, such as residual dsDNA content in raw fish skin (RFS) versus AFS (Section 4.3), an unpaired two-tailed Welch’s *t*-test was used, which does not assume equal variances between groups.

In the porcine wound model, each wound was treated as the experimental unit. Each animal received all three treatments, with two wounds randomly allocated to each group, so that between-animal variability was balanced across the groups; however, the statistical analysis did not account for the clustering of wounds within animals. For comparisons among three or more groups, including re-epithelialization, normalized wound tattoo area, transepidermal water loss, and stratum corneum hydration among the AFS, comparator, and untreated groups at each time point (Section 4.12.3 and Section 4.12.4), and total and type I collagen area among the AFS, comparator, untreated, and normal skin groups (Section 4.12.5), one-way analysis of variance (ANOVA) was performed for parametric data, followed by Dunnett’s multiple comparisons test to compare each treatment group with the untreated group (or with normal skin, where applicable) when a statistically significant overall difference was detected. For non-parametric data, the Kruskal–Wallis H test was used, followed by the Mann–Whitney U test for pairwise comparisons when significant differences were detected. Because each time point was analyzed separately, this approach did not account for the correlation of repeated measurements from the same wound over time or adjust for multiple comparisons across time points.

The semi-quantitative histopathological scores summarized in Table 1 (epithelial status, foreign material/hemorrhage, angiogenesis, hemorrhage severity, and granulation tissue formation/fibroplasia and inflammatory cells) are ordinal data and were therefore analyzed using the Kruskal–Wallis H test, followed by the Mann–Whitney U test for pairwise comparisons when significant differences were detected.

For all analyses, a *p*-value of less than 0.05 was considered statistically significant.

## 5. Conclusions

In this study, an intact, solid-state acellular dermal matrix was prepared from Japanese amberjack skin, an abundant marine byproduct, through a decellularization and decolorization process. The process reduced residual dsDNA to 29.2 ± 7.6 ng/mg dry weight, below the commonly applied threshold of 50 ng/mg, while preserving a collagen-rich (434.1 ± 10.4 µg/mg), structurally asymmetric ECM. The resulting AFS rapidly hydrated, reaching a swelling ratio of 321.3 ± 75.2% within 1 min, and showed a tensile strength of 2.4 ± 0.4 MPa, an elongation at break of 59.6 ± 11.3%, and an initial Young’s modulus of 0.8 ± 0.3 MPa in the hydrated state. AFS was cytocompatible (cell viability, 80.3 ± 4.3%) and elicited only a mild and transient local tissue response with gradual integration over 8 weeks of subcutaneous implantation. In a porcine full-thickness wound model, AFS-treated wounds showed greater re-epithelialization than untreated wounds on Day 7 (25.0 ± 9.2% vs. 5.5 ± 3.1%) and less early expansion of the periwound tattooed area (101.6 ± 6.5% vs. 118.1 ± 11.0% of baseline). At Day 42, they also showed larger collagen-stained (65.2 ± 11.7% vs. 49.7 ± 6.5%) and collagen I-positive (6.8 ± 1.7% vs. 2.9 ± 1.1%) areas. No significant differences from a commercially available cod skin-derived wound matrix were detected across the examined endpoints under the conditions of this study. These findings support the potential of amberjack skin as a marine-derived source of wound-covering matrices. Further studies in larger cohorts and compromised wound models, with adequately powered comparisons and longer-term assessment of scar quality and dermal durability, will be needed to define its clinical potential.

## Figures and Tables

**Table 1:** Histopathological scoring criteria used to evaluate wound healing and tissue responses.

Parameter	Criterion	Score	Parameter	Criterion	Score
**Epithelial status**	None	0	**Foreign material** **/hemorrhage**	Absent	0
	Partial epidermis	1		Present	1
	100% with hyperplasia	2			
	100% normal epidermis	3			
**Angiogenesis**	None	0	**Hemorrhage** **severity**	Absent	0
	≤50 vessels	1		Mild	1
	51–100 vessels	2		Moderate	2
	101–150 vessels	3		Severe	3
	≥151 vessels	4			
**Granulation tissue formation** **/fibroplasia**	None	0	Inflammatory cells *	None	0
	Minimal	1		Minimal	1
	Mild	2		Mild	2
	Moderate	3		Moderate	3
	Marked	4		Marked	4
	Severe	5		Severe	5

* Inflammatory cells include neutrophils, eosinophils, lymphocytes, and macrophages.

## Data Availability

The original contributions presented in this study are included in the article. Further inquiries can be directed to the corresponding author.

## Supplementary Materials

The following supporting information can be downloaded at https://www.mdpi.com/article/10.3390/md24100360/s1.

## Abbreviations

The following abbreviations are used in this manuscript:

ADM	Acellular dermal matrix
AFS	Acellular fish skin
ATR-FTIR	Attenuated total reflectance–Fourier transform infrared spectroscopy
DFU	Diabetic foot ulcer
dsDNA	Double-stranded DNA
ECM	Extracellular matrix
EO	Ethylene oxide
FBS	Fetal bovine serum
H&E	Hematoxylin and eosin
IACUC	Institutional Animal Care and Use Committee
IHC	Immunohistochemistry
ISO	International Organization for Standardization
MEM	Minimum Essential Medium
MT	Masson’s trichrome
OCT	Optimal cutting temperature
PUFA	Polyunsaturated fatty acid
RFS	Raw fish skin
SD	Standard deviation (also used as Sprague–Dawley, referring to the rat strain)
SDS	Sodium dodecyl sulfate
sGAG	Sulfated glycosaminoglycan
SEM	Scanning electron microscopy
TEWL	Transepidermal water loss
UTM	Universal testing machine
VLU	Venous leg ulcer
