# Supplementary material for: Biocompatibility and Wound-Healing Performance of an Amberjack (Seriola quinqueradiata) Skin-Derived Acellular Dermal Matrix in a Porcine Full-Thickness Wound Model
Source: Mar Drugs. 2026 Oct 17;24(10):360. doi: 10.3390/md24100360 (PMC13706585; doi:10.3390/md24100360)
Supplement: Supplementary file 1 [file marinedrugs-24-00360-s001.zip › marinedrugs-4564866-supplementary.pdf]

Epithelial Status										
	Day 7		Day 14		Day 21		Day 28		Day 42	
	Score	N	Score	N	Score	N	Score	N	Score	N
AFS	1	1	1	1	1	1	1	1	3.0 ± 0.0	4
Comparator	1	1	1	1	1	1	1	1	2.8 ± 0.5	4
Untreated	0	1	1	1	1	1	1	1	2.5 ± 0.6	4

Foreign Material/Hemorrhage										
	Day 7		Day 14		Day 21		Day 28		Day 42	
	Score	N	Score	N	Score	N	Score	N	Score	N
AFS	1	1	1	1	0	1	0	1	0.0 ± 0.0	4
Comparator	1	1	0	1	0	1	0	1	0.3 ± 0.5	4
Untreated	0	1	0	1	0	1	0	1	0.0 ± 0.0	4

Angiogenesis										
	Day 7		Day 14		Day 21		Day 28		Day 42	
	Score	N	Score	N	Score	N	Score	N	Score	N
AFS	4	1	4	1	4	1	4	1	2.8 ± 0.5	4
Comparator	4	1	4	1	4	1	4	1	2.8 ± 0.5	4
Untreated	4	1	4	1	4	1	4	1	3.0 ± 0.0	4

Hemorrhage Severity										
	Day 7		Day 14		Day 21		Day 28		Day 42	
	Score	N	Score	N	Score	N	Score	N	Score	N
AFS	3	1	2	1	1	1	2	1	0.0 ± 0.0	4
Comparator	3	1	0	1	1	1	2	1	0.3 ± 0.5	4
Untreated	2	1	1	1	0	1	0	1	0.5 ± 0.6	4

Granulation Tissue Formation and Fibroplasia										
	Day 7		Day 14		Day 21		Day 28		Day 42	
	Score	N	Score	N	Score	N	Score	N	Score	N
AFS	4	1	5	1	5	1	5	1	3.5 ± 1.3	4
Comparator	4	1	4	1	5	1	5	1	3.3 ± 1.3	4
Untreated	5	1	5	1	5	1	5	1	4.3 ± 1.0	4

Neutrophils, Eosinophils, Lymphocytes, and Macrophages										
	Day 7		Day 14		Day 21		Day 28		Day 42	
	Score	N	Score	N	Score	N	Score	N	Score	N
AFS	5	1	3	1	2	1	2	1	1.5 ± 0.6	4
Comparator	4	1	4	1	3	1	2	1	1.5 ± 0.6	4
Untreated	4	1	2	1	2	1	3	1	1.5 ± 0.6	4

Supplementary Figure S1. Semi-quantitative histopathological scoring of wound healing based on H&E-stained sections. Wounds treated with AFS or the comparator (Kerecis Omega3 Wound), or left untreated, were scored on Days 7, 14, 21, 28, and 42 for epithelial status, foreign material/hemorrhage, angiogenesis, hemorrhage severity, granulation tissue formation and fibroplasia, and inflammatory cell infiltration (neutrophils, eosinophils, lymphocytes, and macrophages), using a previously described scoring system [50]. Scores for Days 7–28 were obtained from a single animal dedicated to histopathological analysis (n = 1 per group at each time point) and are presented as individual scores without statistical comparison. Scores for Day 42 were obtained from two animals (n = 4 wounds per group) and are presented as the mean ± SD; no statistically significant differences among the groups were detected for any parameter (Kruskal–Wallis H test). AFS, acellular fish skin; H&E, hematoxylin and eosin.
